# Family Preference for Place of Death Mediates the Relationship between Patient Preference and Actual Place of Death: A Nationwide Retrospective Cross-Sectional Study

**DOI:** 10.1371/journal.pone.0056848

**Published:** 2013-03-08

**Authors:** Yoshiki Ishikawa, Sakiko Fukui, Toshiya Saito, Junko Fujita, Minako Watanabe, Kazuhiro Yoshiuchi

**Affiliations:** 1 Department of Public Health, Jichi Medical University, Shimotuke-shi, Tochigi, Japan; 2 Cancer Scan, Shibuya-ku, Tokyo, Japan; 3 Department of Community Health Nursing, Graduate School of Nursing, The Japanese Red Cross University, Shibuya-ku, Tokyo, Japan; 4 Department of Nursing, Ibaraki Christian University, Hitachi-shi, Ibaraki, Japan; 5 Department of Stress Sciences and Psychosomatic Medicine, Graduate School of Medicine, The University of Tokyo, Bunkyo-ku, Tokyo, Japan; Universidad Europea de Madrid, Spain

## Abstract

**Background:**

Discrepancy between preferred and actual place of death is common in cancer patients. While previous research has elucidated the factors associated with congruence between patients' preferred and actual place of death, it is not known how the perspective of the family influences the place of death. This study examined whether family preference for place of death mediates the relationship between patient preference and actual place of death.

**Methods:**

A total of 258 cancer patients (home death, n = 142; hospital death, n = 116) who had received terminal care in Japan were analyzed. Measures included patients' demographic variables, patient and family preferences for place for death, actual place of death, patients' functional status, use and intensity of home care, availability of inpatient bed, living arrangement, and amount of extended family support.

**Results:**

Patient-family congruence on preferred place of death was 66% in patients who died at home and 47% in patients who died at other places (kappa coefficient: 0.20 and 0.25, respectively). In a multiple logistic regression model, patients were more likely to die at home when patients were male (odds ratio [OR], 95% confidence interval [CI]: 2.53, 1.06–6.05) and when their family preferred death at home (OR, 95% CI: 37.37, 13.82–101.03). A Sobel test revealed that family preference mediated the relationship between patient preference and place of death (p<0.05).

**Conclusions:**

This study is, to our knowledge, the first to unveil the role of the family in the relationship between patient preference and place of death in Japan. In order to honor patients' wishes to die at home, supporting caregivers in the family may be an essential component of terminal care.

## Introduction

Discrepancies between preferred and actual place of death is common [1], [2]. For example, most of the terminally ill cancer patients who have indicated a preference to die at home actually die in hospitals [3], [4], [5], [6]. A previous study reported that cancer patients who died in a hospital or intensive care unit are more likely to have a lower quality of life at their end-of-life compared with those who die at home [7]. Thus, bridging the gap between preferred and actual place of death is one essential component of terminal care for cancer patients.

Past research has systematically elucidated the factors associated with congruence between preferred and actual place of death, including symptom control, physician support, hospice enrollment, and family support [1]. Specifically, the family plays an important role in the final decision making on the place of death through negotiation with the patient [8]. Another research has shown how family caregivers are more likely than patients to prefer hospitals as the place for death [4]. In addition, regardless of the wish of patients, a family preference for hospital death is negatively correlated with death at home [9]. Given the complex nature of the mechanisms concerning places of death, further insights on perspectives of the family is needed. However, at present, few studies have examined how patient and family preferences for place of death interact and influence the actual place of death in a joint manner.

In this study we utilized nationwide cross-sectional data from Japan to examine whether family preference for place of death mediates the relationship between patient preference and actual place of death. It is hoped that the findings from our study will provide directions for the implementation of more effective policies or design of intervention programs to address the gap between patients' preferred and actual place of death.

## Materials and Methods

### Data Source

Data from the 2011 Terminal Care and Team Collaboration Survey by the Ministry of Health, Labour and Welfare of Japan (Ministry of Health, Labour and Welfare, 2011) were used for analysis in this study. This survey was originally a nationwide cross-sectional retrospective survey on current trends of terminal care and physician-nurse collaboration in Japan, and was conducted in April 2011. The variables used in this study are described in the ‘Measures’ section. Eight hundred and eighty eight home visit nursing agencies from a total of 5074 agencies in Japan were first randomly selected from the Welfare and Medical Service Network System database. A questionnaire was then distributed to primary nurses at these selected agencies, requiring responses regarding the two most recent patients with the following criteria: (1) those who had received terminal care for more than one month, and (2) who had died at home or in a hospital. The primary nurse for each eligible patient was requested to complete the self-administered questionnaire by reviewing patient records retrospectively.

### Measures

The measures used for analysis in this present study were selected based on systematic reviews of the literature on the potential determinants of the place of death [8], [10], including demographic variables of patients, patient and family preferences for place for death, actual place of death, patients' functional status, use and intensity of home care, availability of inpatient bed, living arrangement, and the amount of extended family support. Table 1 presents the variables analyzed in this study.

**Table 1:** Frequencies and percentages of analyzed variables.

		Home death (n = 142)	Hospital death (n = 116)
Demographic variables			
Sex			
Male		81 (57)	60 (52)
Female		59 (42)	55 (47)
Missing		2 (1)	1 (1)
Age			
–59		5 (4)	6 (5)
60–69		10 (7)	13 (11)
70–79		46 (32)	41 (35)
80–89		57 (40)	42 (36)
90–		19 (13)	7 (6)
Missing		5 (4)	7 (6)
Individual factors			
Patient preference for place of death			
Home		93 (65)	30 (26)
Other places/Unsure		46 (32)	81 (70)
Missing		3 (2)	5 (4)
Factors related to illness			
Functional status of patients			
Bedridden		98 (69)	61 (53)
Half-bedridden		36 (25)	45 (39)
Independent		6 (4)	7 (6)
Missing		2 (1)	3 (3)
Environmental factors			
Intensity of physician visit one month before death or admission (visits/month)			
More than 5 times		39 (27)	10 (9)
Less than 4 times		90 (63)	88 (76)
Missing		13 (9)	18 (16)
Intensity of nurse visit one month before death or admission (visits/month)			
More than 13 times		73 (51)	32 (28)
Less than 12 times		57 (40)	65 (56)
Missing		12 (8)	19 (16)
Availability of inpatient beds			
Available		23 (16)	6 (5)
Not Available		106 (75)	106 (91)
Missing		8 (6)	1 (1)
Number of available family support			
More than 2		32 (23)	25 (22)
One		94 (66)	18 (16)
None		14 (10)	67 (58)
Missing		2 (1)	6 (5)
Family preference for place of death			
Home		117 (82)	13 (11)
Other places/Unsure		22 (15)	99 (85)
Missing		3 (2)	4 (3)

### Ethics Statement

Ethics approval for this study was obtained from the Institutional Review Board of The Japanese Red Cross College of Nursing in 2010.

### Statistical Analysis

The frequencies and percentages for each major study variable were first obtained. Bivariate and multiple logistic regression analyses using home death as the dependent variable were then conducted. In addition, the kappa coefficient [11] was calculated to examine the patient-family congruence on preferred place of death. The defining criteria for the strength of the congruence were as follows: ≦0.20, poor; 0.21–0.40, fair; 0.41–0.60, moderate; 0.61–0.80, substantial; 0.81–1.00, almost perfect [12]. Finally, a Sobel test [13] was conducted to examine whether family preference mediates the relationship between patient preference and actual place of death. We adopted a 5%, two-tailed significance level. SAS 9.2 (SAS Institute, Cary, NC) was used for all statistical analyses.

## Results

Out of 1776 questionnaires that were distributed, a total of 396 questionnaires were completed, corresponding to a response rate of 34.2%. Two hundred and fifty eight of these 396 patients had cancer as the primary cause of death, and were extracted for analysis in this study.

### Descriptive data

Table 1 shows the descriptive statistics of the variables used in this study. Patient and family preference for home death were 47% (n = 123) and 50% (n = 130), respectively.

### Congruence between patient and family preference for place of death

Table 2 and 3 shows the family-patient congruence on preferred place of death. The kappa coefficient of congruence in patients who died at home was 0.20 (95% confidence interval [CI]: 0.07–0.33), indicating a poor congruence between patient and family preference for place of death. The kappa coefficient of congruence in patients who died at other places was 0.25 (95% confidence interval [CI]: 0.14–0.37), indicating a fair association between patient and family preference for place of death.

**Table 2:** Patient-family congruence on preferred place of death among those who died at home.

Patient preference for place of death	Family preference for place of death			
	Home	Other places	Unknown	Total
Home	83	8	1	92
Other Places	3	7	0	10
Unknown	29	5	1	35
Total	115	20	2	137

**Table 3:** Patient-family congruence on preferred place of death among those who died at other places.

Patient preference for place of death	Family preference for place of death			
	Home	Other places	Unknown	Total
Home	7	22	0	29
Other Places	1	45	0	46
Unknown	5	22	8	35
Total	13	89	8	110

### Bivariate and Multiple logistic regression analyses for home death

The results of bivariate and multiple logistic regression analyses for home death are appended in Table 4. Patients were more likely to die at home when they were male (odds ratio [OR], 95% CI: 2.53, 1.06–6.05), and when their families preferred home death (OR, 95% CI: 37.37, 13.82–101.03). Age, preference for place for death, functional status, use and intensity of home care, availability of inpatient bed, living arrangement, and amount of extended family support were not related to home deaths.

**Table 4:** Bivariate and multiple logistic regression analyses for home death.

Demographic variables		
Sex		
Male	1.44 (0.86–2.39)	2.53 (1.06–6.05)
Female	reference	reference
Age		
–59	0.28 (0.06–1.26)	0.40 (0.05–3.32)
60–69	0.26 (0.08–0.89)	0.83 (0.13–5.18)
70–79	0.38 (0.14–1.04)	1.45 (0.34–6.22)
80–89	0.47 (0.17–1.29)	0.97 (0.24–3.93)
90–	reference	reference
Individual factors		
Patient preference for place of death		
Home	5.71 (3.28–9.94)	2.18 (0.95–5.03)
Other places/Unsure	reference	reference
Factors related to illness		
Functional status of patients		
Independent	0.62 (0.19–2.02)	0.21 (0.03–1.24)
Half-bedridden	0.49 (0.29–0.86)	0.82 (0.35–1.92)
Bedridden	reference	reference
Environmental factors		
Intensity of physician visit one month before death or admission (visits/month)		
More than 5 times	3.32 (1.59–6.95)	2.03 (0.68–6.07)
Less than 4 times	reference	reference
Intensity of nurse visit one month before death or admission (visits/month)		
More than 13 times	2.54 (1.47–4.41)	1.02 (0.42–2.48)
Less than 12 times	reference	reference
Availability of inpatient beds		
Available	0.23 (0.08–0.66)	0.60 (0.15–2.43)
Not Available	reference	reference
Number of available family support		
None	0.07 (0.01–0.68)	1.33 (0.16–11.15)
One	0.31 (0.16–0.61)	0.60 (0.21–1.76)
More than 2	reference	reference
Family preference for place of death		
Home	39.0 (18.67–81.5)	37.37 (13.82–101.03)
Other places/Unsure	reference	reference

OR: Odds Ratio, CI: Confidence Interval

### The mediating effect of family preference for place of death

**Figure 1:**
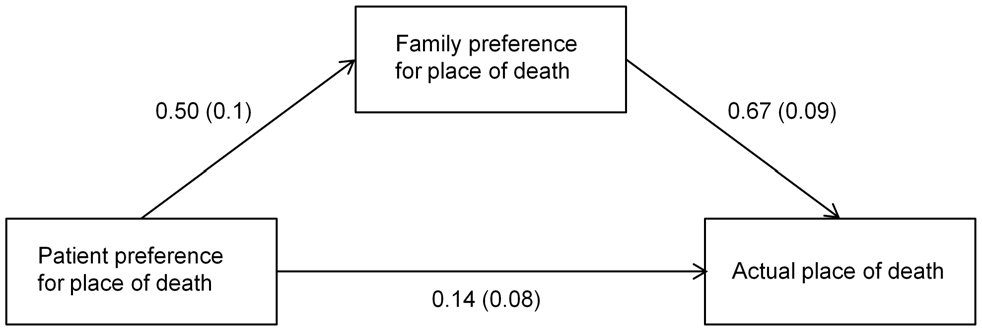
Family preference as a mediator of the association between patient preference and place of death. The number shows coefficient and standard deviation in parentheses. There is a significant relationship between patient preference and actual place of death. Once family preference is added in, the relationship between patient preference and actual place of death shown in Figure 1 drops to non-significant levels. This shows that family preference is a mediator of the relationship between patient preference and actual place of death.

Figure 1 depicts the result of the Sobel test, indicating that family preference for place of death is a significant mediator (p<0.05). The direct effect of patient preference for place of death on the actual place of death is significantly attenuated by family preference (Table 4).

## Discussion

While substantial efforts have been undertaken to enhance the quality of home care worldwide [14], [15], [16], most terminal cancer patients die in hospitals despite their preferences to die at home [3], [4], [5], [6]. Past research suggests that dying at home increase both patients and families' quality of life [7]. It is thus important to further understand the mechanisms behind the relationship between preferred and actual place of death so as to guide the implementation of more effective policies or design of intervention programs in this area. To our knowledge, this is the first nationwide study to unveil the role of a family perspective in the relationship between patient preference and place of death.

The most important finding of the present study is how family preference for place of death mediates the relationship between patient preference and place of death. This result is an important contribution to research on mechanisms explaining preferred and actual places of death. Although some studies have examined the mediation effect of family preference for place of death, they have mainly focused on examining the direct effect of patient and family preference on actual place of death [5], [6], [8], [9]. What our study has shown however is the indirect effect patient preference has on the actual place of death rather than a direct effect. This pathway may be partly explained by a unique culture of a group-oriented sense of self and decision making in Japan [17], as beliefs, norms and tradition of care-giving have been reported to be largely influenced by cultural factors [18], [19], [20]. Further studies focusing on the cultural context of care giving during one's end-of-life will enable us to understand better the mechanisms of how patient and family preferences interact and jointly influence the place of death.

Another important finding of this study is that low congruence between patient and family preference for place of death was observed. Congruence between patients' and their families' preferred place of death is one of the important goals in terminal care. Patient-family congruence on the preferred place of death is reported to be positively associated with improved quality of life in terminally ill cancer patients [21]. A systematic review has shown that symptom control, physician support, hospice enrollment, and family support are factors that influence congruence between patient preference and place of death [1]. However, studies from Asia are less representative in this review and further research on the factors influencing congruence is therefore needed.

This study has several limitations. First, causality cannot be inferred from this study because cross-sectional data were used. Further longitudinal studies are required to examine whether family preference for place of death mediates the relationship between patient preference and actual place of death. Second, the response rate of home nursing agencies was low (34.2%), therefore the result of this study cannot be generalized throughout Japan. There might be a couple of reasons why the response rate was low: 1) some home nursing agencies did not meet the inclusion criteria for this study, for example, they did not have patient who was provided terminal care services during a study duration. According to our previous nationwide survey for home nursing agencies [22], 39% of home nursing agencies did not provide terminal care services in 2011: 2) nursing home agencies had only two weeks to return the survey, so that this might lower the response rate; and 3) the burden of filling out the A4-sized 10 pages of the questionnaire for each patient case might discourage the study participating agencies to return the questionnaire. Third, the validity of the data used in this study requires further exploration, because all data were collected by primary nurses based on patients' medical records retrospectively. Finally, not all confounders associated with place of death were accounted for. However, major confounders identified in a previous systematic review were controlled statistically, thereby reducing chances of producing bias when examining the mediating role of family preference.

In summary, this study found that family preference for place of death mediates the relationship between patient preference and actual place of death. Further research on the mechanism concerning the place of death while considering the role of the family will contribute to developing quality terminal care programs or policies in Japan.
